# Supplementary material for: Induction and regulation of reversible suspended animation in C. elegans
Source: Nat Commun. 2026 Mar 31;17:4627. doi: 10.1038/s41467-026-71247-9 (PMC13199508; doi:10.1038/s41467-026-71247-9)
Supplement: Supplementary file 1 — Supplementary Information [file 41467_2026_71247_MOESM1_ESM.pdf]

Induction and regulation of a reversible form of suspended animation in *C. elegans*

Junqiang Liu[1], Bingying Wang[1], Jonathan Leon Catrow[2], Quentinn Pearce[2], Zhijian Ji[1], Supeng Winnie Yang[1], Akash Balakrishnan[1], James E. Cox[2], Dengke K. Ma[1, 3, 4, *]

[1]Cardiovascular Research Institute, University of California San Francisco, San Francisco, CA, USA.

[2]Metabolomics Core Research Facility, Department of Biochemistry, University of Utah, Salt Lake City, Utah, USA.

[3]Department of Physiology, University of California San Francisco, San Francisco, CA, USA.

[4]Innovative Genomics Institute, Berkeley, CA, USA.

* Corresponding author. Email: Dengke.Ma@ucsf.edu

Supplementary Figures and Legends

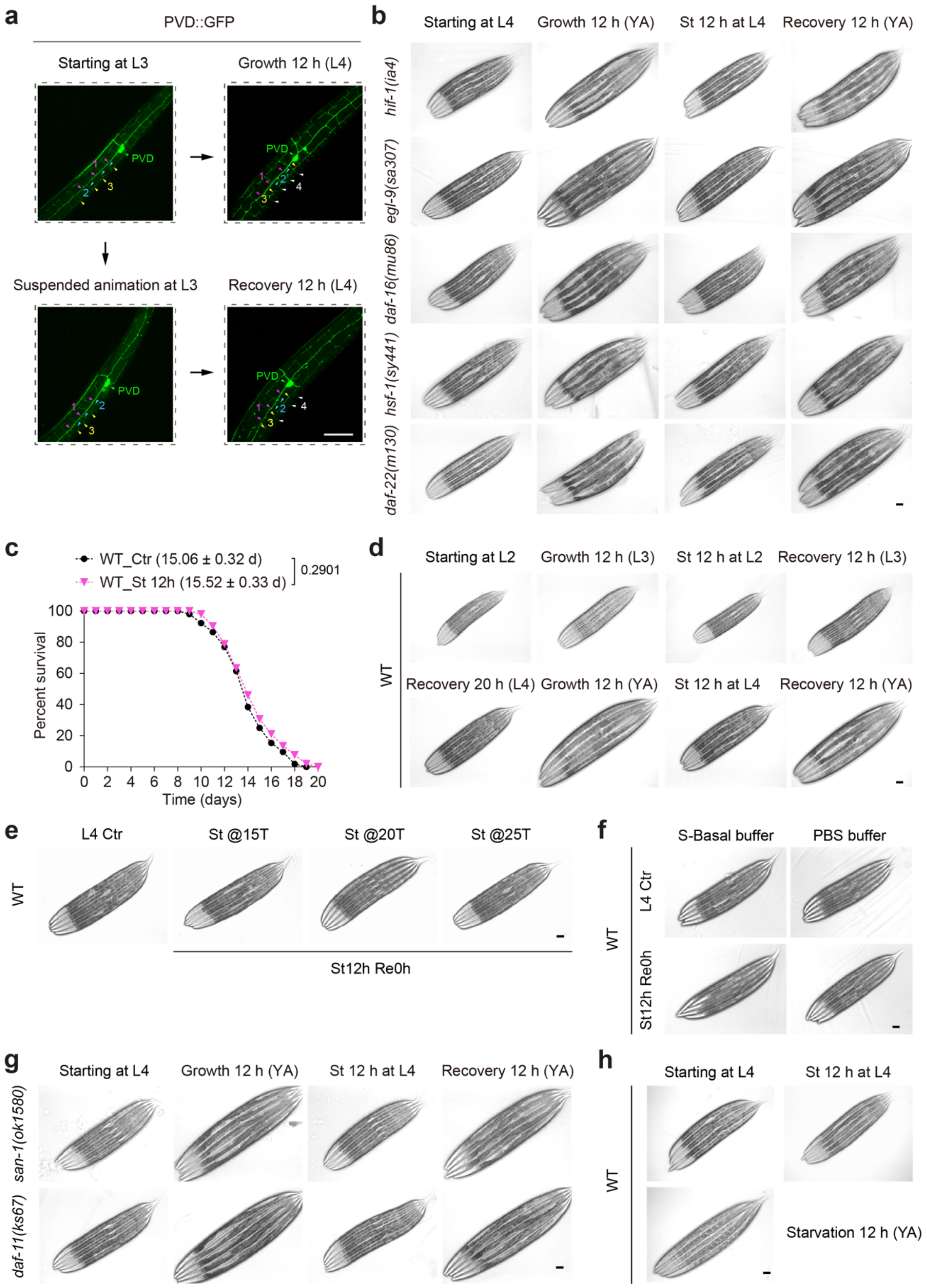

Supplementary Fig. 1 Characterization of physiological properties of LISA. a, Representative fluorescence images of *C. elegans* PVD::GFP reporters, LISA-induced arrest in the 3rd dendrite branching (*n* = 15 worms). **b**, Representative brightfield images of L4-stage and adult animals, showing normal LISA induction in wild type, *hif-1*, *egl-9*, *daf-16*, *hsf-1* and *daf-22* mutants (*n* = 20 worms in each of 3 independent experiments). **c**, Representative lifespan curves of *C. elegans*, showing no apparent effect of larval LISA on adult lifespans (*n* = 55 worms per test). *P* values are shown. Three independent experiments were performed; mean lifespan (± s.d.) and representative lifespan curves are shown. **d**, Representative brightfield images of larval-stage and adult animals, showing normal LISA induction following earlier LISA in wild type (*n* = 15 worms in each independent experiment). **e**, Representative brightfield images showing normal LISA induction at various indicated temperatures (*n* = 15 worms in each independent experiment). **f**, Representative brightfield images showing normal LISA induction with indicated isotonic buffers (*n* = 15 worms in each independent experiment). **g**, Representative brightfield images showing normal LISA induction in *san-1* or *daf-11* mutants (defects in anoxia-induced LISA and dauer diapause, respectively) (*n* = 15 worms in each independent experiment). **h**, Representative brightfield images showing normal LISA induction with food deprived during and after standing (*n* = 15 worms in each independent experiment). In **c**, the Kaplan–Meier method followed by the log-rank test was used. Scale bars, 50 μm (**a**, **b**, **d**–**h**).

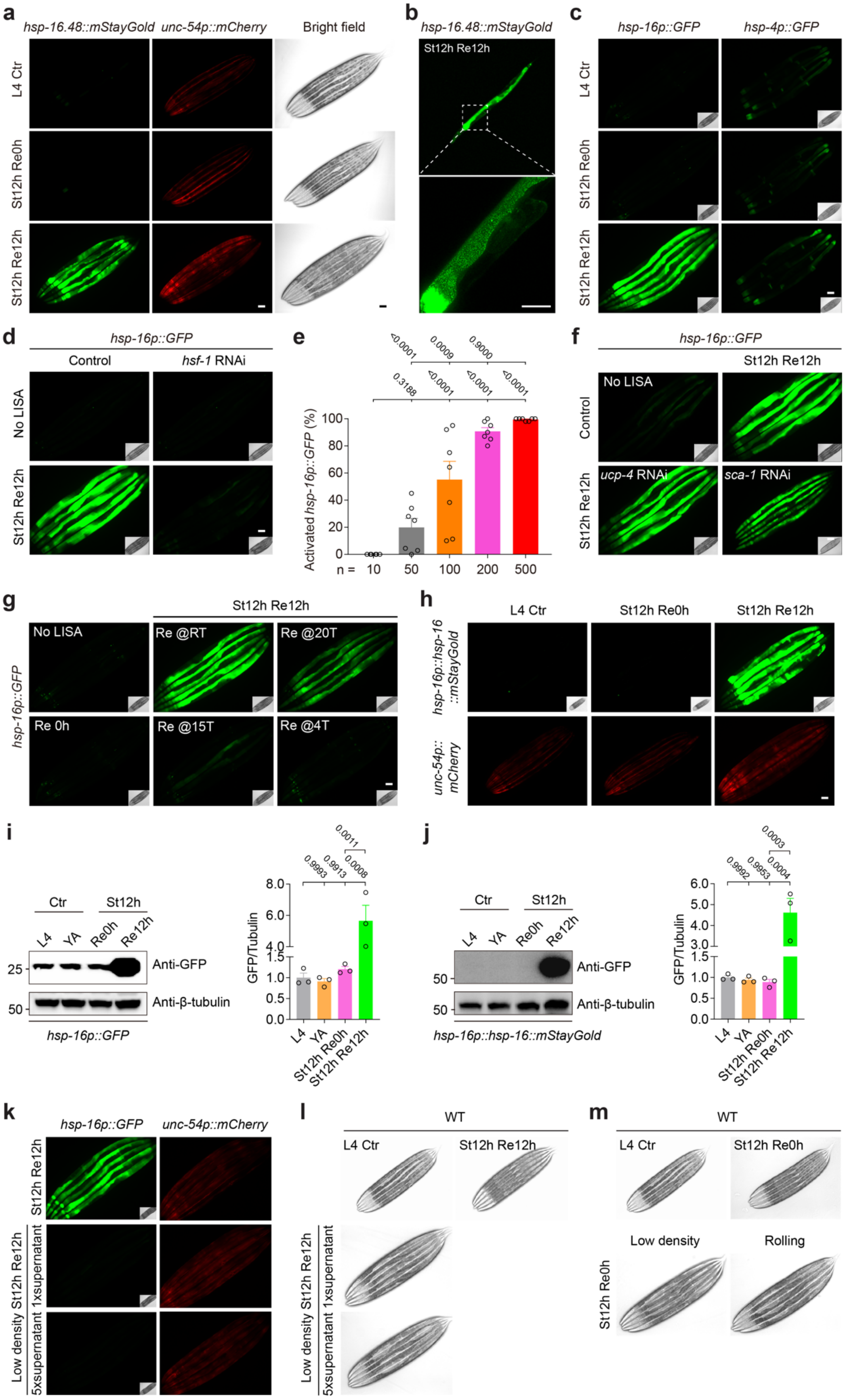
a
hsp-16.48::mStayGold
unc-54p::mCherry
Bright field
L4 Ctr
St12h Re0h
St12h Re12h
b
hsp-16.48::mStayGold
St12h Re12h
c
hsp-16p::GFP
hsp-4p::GFP
L4 Ctr
St12h Re0h
St12h Re12h
d
hsp-16p::GFP
Control
hsf-1 RNAi
No LISA
St12h Re12h
e
Activated hsp-16p::GFP (%)
<0.0001
0.0009
0.9000
0.3188
<0.0001
<0.0001
<0.0001
100
80
60
40
20
0
n =
10
50
100
200
500
f
hsp-16p::GFP
St12h Re12h
No LISA
Control
ucp-4 RNAi
sca-1 RNAi
St12h Re12h
g
St12h Re12h
No LISA
Re @RT
Re @20T
hsp-16p::GFP
Re 0h
Re @15T
Re @4T
h
L4 Ctr
St12h Re0h
St12h Re12h
hsp-16p::hsp-16::mStayGold
unc-54p::mCherry
i
Ctr
St12h
L4
YA
Re0h
Re12h
25
Anti-GFP
50
Anti-β-tubulin
hsp-16p::GFP
GFP/Tubulin
0.0011
0.9993
0.9913
0.0008
8.0
6.0
4.0
1.5
1.0
0.5
0.0
L4
YA
St12h Re0h
St12h Re12h
j
Ctr
St12h
L4
YA
Re0h
Re12h
50
Anti-GFP
50
Anti-β-tubulin
hsp-16p::hsp-16::mStayGold
GFP/Tubulin
0.0003
0.9992
0.9953
0.0004
6.0
5.0
4.0
3.0
1.5
1.0
0.5
0.0
L4
YA
St12h Re0h
St12h Re12h
k
hsp-16p::GFP
unc-54p::mCherry
St12h Re12h
Low density St12h Re12h
1xsupernatant
5xsupernatant
l
WT
L4 Ctr
St12h Re12h
Low density St12h Re12h
1xsupernatant
5xsupernatant
m
WT
L4 Ctr
St12h Re0h
Low density
Rolling
St12h Re0h

Supplementary Fig. 2. Characterization of *hsp-16p::GFP* transcriptional and translational reporter upregulation by LISA. a, Representative fluorescence images of *C. elegans hsp-16*p::*hsp-16::GFP* translational reporters, confirming drastic upregulation by LISA after recovery from LISA, but not during LISA (n = 15 worms in each of 3 independent experiments). **b**, Enlarged view of *hsp-16*p::*hsp-16::GFP*, showing discrete fluorescent puncta indicated (n = 10). **c**, Representative fluorescence images of *C. elegans* transcriptional reporters, showing drastic upregulation *hsp-16p::GFP* but not *hsp-4p::GFP* by LISA after recovery from LISA (n = 15 worms in each of 3 independent experiments). **d**, Representative fluorescence images of *C. elegans hsp-16p::GFP* transcriptional reporters, showing drastic upregulation after recovery from LISA in control but suppressed in *hsf-1* RNAi animals (n = 20 worms in each of 3 independent experiments). **e**, Quantification of *hsp-16p::GFP* upregulation after LISA, showing population density dependency as for LISA. **f**, LISA-induced *hsp-16p::GFP* upregulation in control but not suppressed in *ucp-4* or *sca-1* RNAi animals (n = 15 worms in each of 3 independent experiments). **g**, Representative fluorescence images showing LISA-induced *hsp-16p::GFP* upregulation in 20 °C or 25 °C but suppressed at lower (4 °C or 15 °C) temperature (n = 15 worms in each of 3 independent experiments). **h**, Representative fluorescence images showing LISA-induced *hsp-16p::hsp-16::mStayGold* upregulation after standing. **i**, Representative Western blot and quantification results showing LISA-induced *hsp-16p::GFP* upregulation after standing (n > 500 worms in each of 3 independent experiments). **j**, Representative Western blot and quantification results showing LISA-induced *hsp-16p::hsp-16::mStayGold* upregulation after standing (n > 500 worms in each of 3 independent experiments). **k, l,** Representative fluorescence (k) and brightfield (l) images showing normal *hsp-16p::GFP* and LISA induction supplemented with 1x or 5x volumes of supernatants from

animals under LISA (n = 15 worms in each independent experiment). **m,** Representative brightfield images showing normal LISA induction under low density or dispersed rolling conditions (n = 15 worms in each independent experiment). In **e, i, and j**, one-way ANOVA was used to compare all datasets. P values are shown. Scale bars, 50 μm (**a**–**d**,**f**–**h**,**k**–**m**).

a

Significant changes of a.a. and mitochondrial energy metabolites by GC-MS analysis
(P value < 0.05, Fold Change > 1.5, raw adjusted)

Compound	FC	log2(FC)	raw.Pval	-log10(P)
Succinic acid	3.840600	1.941300	0.002654	2.576100
myo-Inositol	2.273200	1.184700	0.002246	2.648600
Spermidine	1.941900	0.957460	0.038917	1.409900
Pyruvate	1.897500	0.924100	0.029787	1.526000
L-Lactic acid	1.712700	0.776280	0.004345	2.362000
Nonanoic acid	0.630680	-0.665030	0.014572	1.836500
L-Serine	0.565100	-0.823420	0.037284	1.428500
L-Aspartic acid	0.484500	-1.106200	0.005052	2.296600
Glycerol 3-phosphate	0.410830	-1.283400	0.000265	3.576100
Ribose	0.288310	-1.794300	0.000562	3.250600
D-Glucose	0.288110	-1.795300	0.000529	3.276800
Malonic acid	0.273280	-1.871600	0.000177	3.752800

b

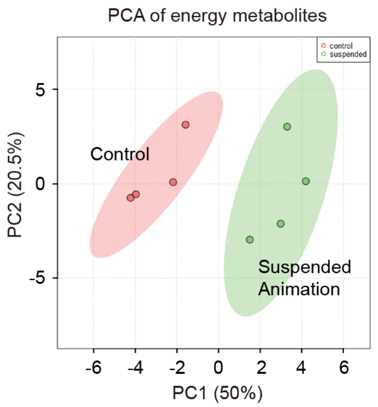

Supplementary Fig. 3. Characterization of LISA-induced metabolomic phenotypes. a, GC-MS metabolomic analysis, showing LISA-induced upregulation of metabolites including succinate and lactate, consistent with a hypometabolic state indicated by LC-MS profiling of energy metabolites. Shown are metabolites with differential abundance by LISA, with an adjusted P value < 0.05 and a fold change > 1.5. **b**, PCA of LISA-regulated metabolites measured by GC-MS. Four biological replicates were included for each treatment (**a**,**b**).

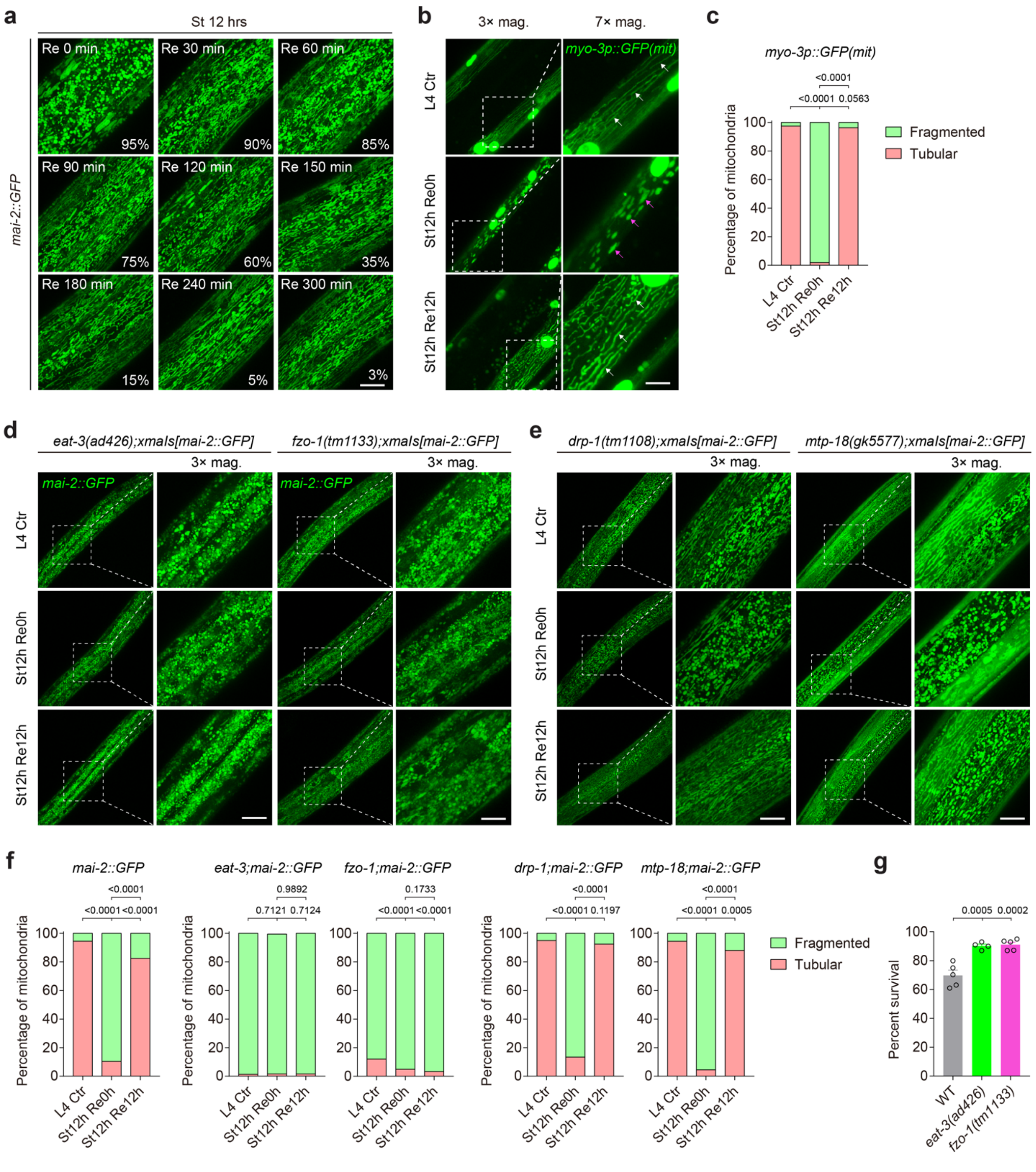

Supplementary Fig. 4. Characterization of LISA-induced mitochondrial phenotypes. a, Representative confocal fluorescence images of *C. elegans mai-2::GFP* translational reporters, showing drastic mitochondrial morphological remodeling in multiple tissues, including intestine, hypodermis and muscles, by LISA and after recovery from LISA (*n* = 5 worms in each of 3

independent experiments). **b**, Representative confocal fluorescence images of *C. elegans myo-3p::mito::GFP* translational reporters, showing drastic mitochondrial morphological remodeling in body wall muscles, by LISA and after recovery from LISA (Left to right, *n* = 18, 20, 20). White and red arrowheads indicate tubular and fragmented mitochondria, respectively. **c,** Quantification of LISA-induced mitochondrial morphological remodeling. **d**, Representative confocal fluorescence images of *C. elegans mai-2::GFP* translational reporters, showing suppressed mitochondrial morphological remodeling in *eat-3* and *fzo-1* mutants by LISA (Left to right, *n* = 6, 7, and 7 (for *eat-3*) and 5, 6, and 5 (for *fzo-1*) in each of 3 independent experiments). **e**, Representative confocal fluorescence images of *C. elegans mai-2::GFP* translational reporters, showing largely normal mitochondrial morphological remodeling in *drp-1* and *mtp-18* mutants by LISA (Left to right, *n* = 5, 6, and 5 (for *drp-1*) and 5, 5, and 5 (for *mtp-18*) in each of 3 independent experiments). **f**, Quantification of mitochondrial remodeling defects of mutants indicated. **g**, Quantification of survival rates of mutants indicated, suggesting a non-essential role of mitochondrial remodeling in surviving LISA (*n* = 400 worms in each independent experiment). Data show mean ± s.e.m. One-way ANOVA with Tukey's multiple comparison test was used (**c**,**f**) or Student's two-tailed unpaired *t*-test was used to compare mutant data with WT (**g**). *P* values are indicated. Scale bars, 50 μm (**a**,**b**,**d**,**e**).

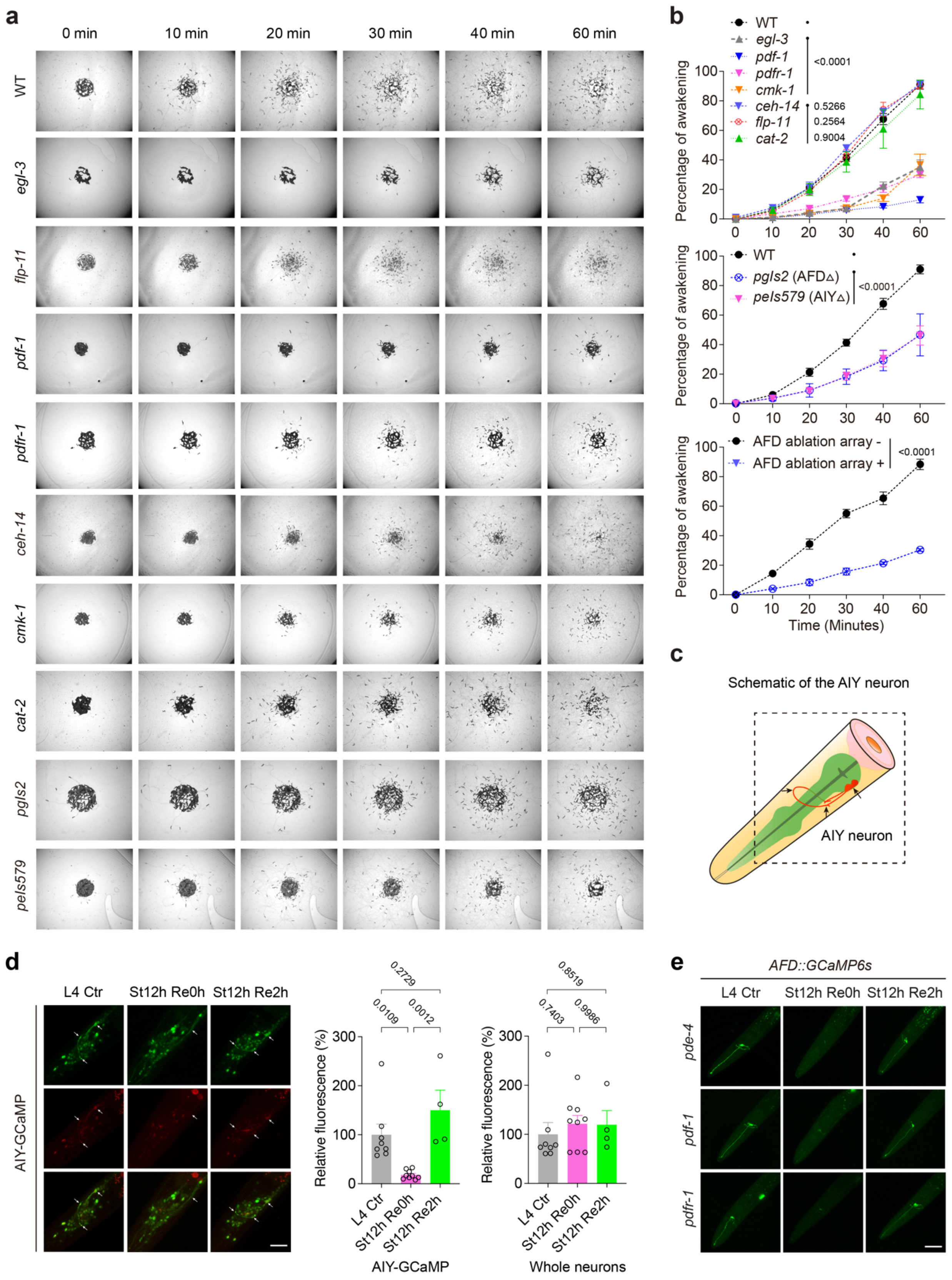

a
0 min
10 min
20 min
30 min
40 min
60 min
WT
egl-3
flp-11
pdf-1
pdfr-1
ceh-14
cmk-1
cat-2
pgIs2
peIs579
b
WT
egl-3
pdf-1
pdfr-1
cmk-1
ceh-14
flp-11
cat-2
<0.0001
0.5266
0.2564
0.9004
Percentage of awakening
pgIs2 (AFD△)
peIs579 (AIY△)
AFD ablation array -
AFD ablation array +
Time (Minutes)
c
Schematic of the AIY neuron
AIY neuron
d
L4 Ctr
St12h Re0h
St12h Re2h
AIY-GCaMP
0.2729
0.0109
0.0012
Relative fluorescence (%)
0.8519
0.7403
0.9986
Whole neurons
e
AFD::GCaMP6s
pde-4
pdf-1
pdfr-1

Supplementary Fig. 5. Characterization of LISA awakening regulation by various neurons and molecules. a, Time-serial brightfield images showing locomotion behavioral recovery post-LISA in wild type and various strains indicated, including AFD, ALA and AIY neuron ablation mutants, neuropeptide mutants and dopamine-deficient *cat-2* mutants. GABA and glutamate neurotransmitter mutants were excluded owing to baseline locomotion defects. **b**, Population quantification of (A) and [*gcy-8p::ICE; myo-2::mCherry*] array+ and array- animals (with or without genetic ablation of AFD, respectively) (*n* = 150 worms in each of 3 independent experiments). **c**, Schematic of AIY neuron. Black arrows indicate the soma and axon. The diagram was generated using Adobe Illustrator (2020). **d**, Representative GCaMP fluorescence images and quantification of time-series calcium activity in AIY neurons and all neurons during LISA and following recovery, demonstrating profound LISA-induced neuronal silencing and reactivation of AIY neurons. Arrows indicate the soma and axon. (Left to right, *n* = 8, 7, and 4). Data show mean ± s.e.m. **e**, Representative AFD::GCaMP fluorescence images for time-serial calcium levels during LISA exit in cAMP-overproducing *pde-4(LOF)* mutants, or PDF signaling defective *pdf-1(LOF)* and *pdfr-1(LOF)* mutants, showing LISA-induced silencing and reactivation of AFD neurons as in wild type (*n* = 5 worms in each of 3 independent experiments). Two-way ANOVA with Bonferroni post-test was used to compare mutant data with WT or worms with or without AFD ablation (**b**) or one-way ANOVA with Tukey's multiple comparison test was used in (**d**). *P* values are indicated. Scale bars, 50 µm (**d**,**e**).

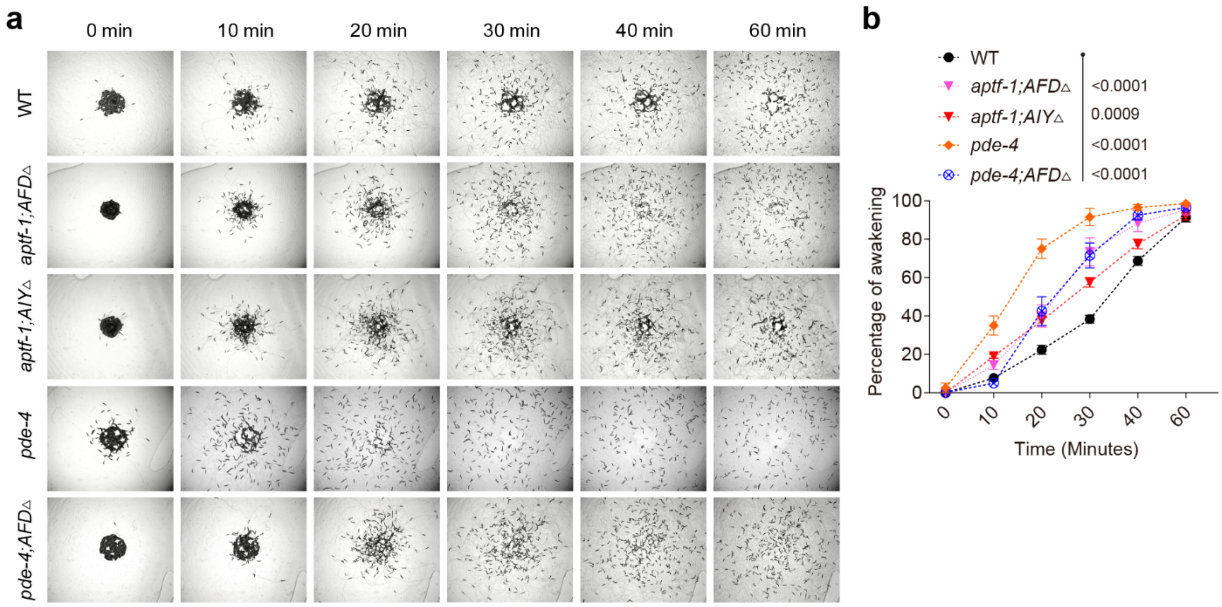

Supplementary Fig. 6. Loss of PDE-4 or the RIS neuron is dominant over the absence of AFD or AIY neurons in promoting awakening. a, Time-serial brightfield images showing behavioral recovery post-LISA in wild type and various strains indicated, including cAMP-overproducing *pde-4* mutants, RIS-deficient *aptf-1* mutants, with the genetic ablation of AFD or AIY using caspase expression. **b**, Population quantification of (A) (n = 150 worms in each of 3 independent experiments). Two-way ANOVA with Bonferroni post-test was used to compare mutants with WT (**b**). P values are indicated.

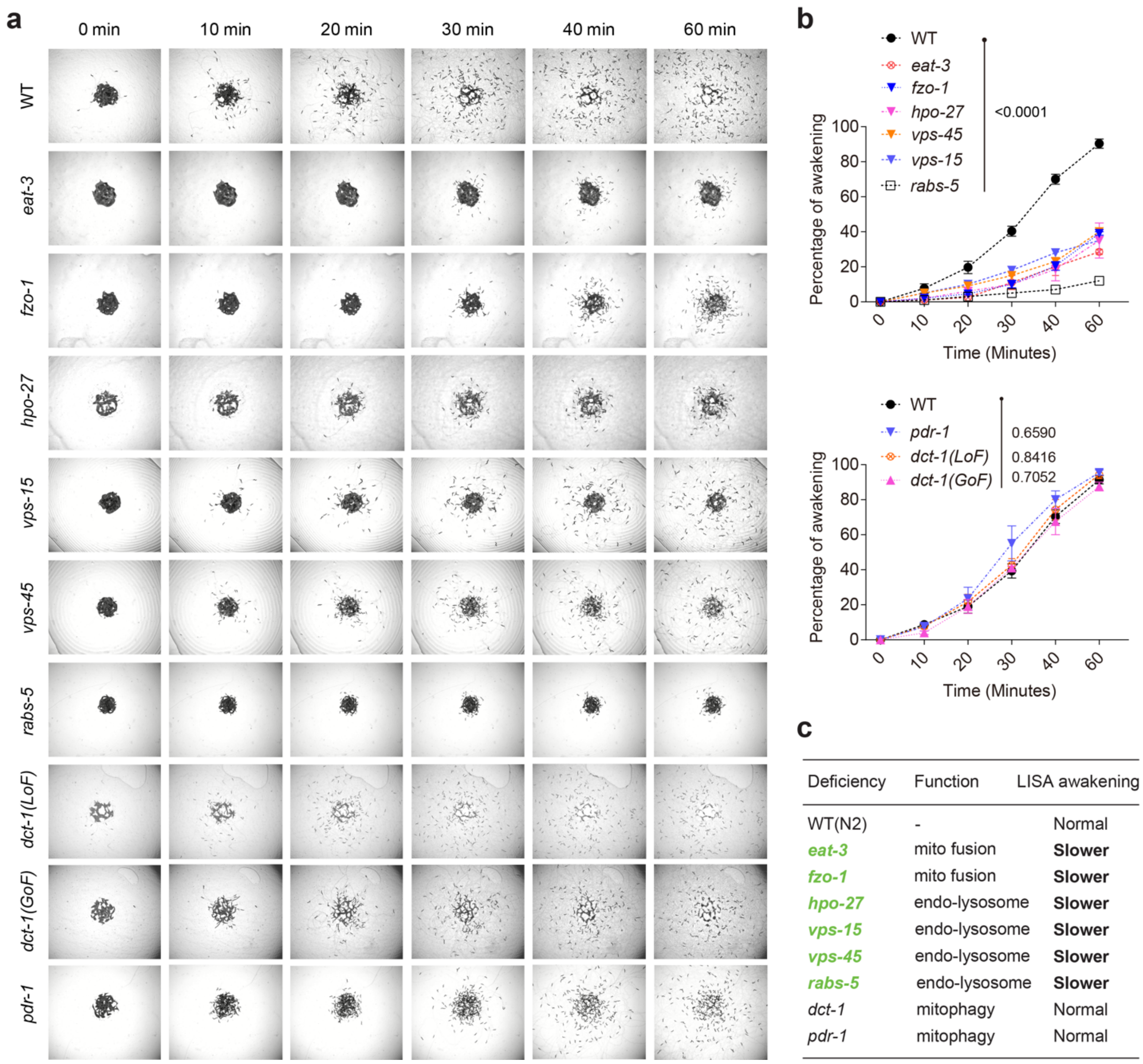

Deficiency	Function	LISA awakening
WT(N2)	-	Normal
eat-3	mito fusion	**Slower**
fzo-1	mito fusion	**Slower**
hpo-27	endo-lysosome	**Slower**
vps-15	endo-lysosome	**Slower**
vps-45	endo-lysosome	**Slower**
rabs-5	endo-lysosome	**Slower**
dct-1	mitophagy	Normal
pdr-1	mitophagy	Normal

Supplementary Fig. 7. Characterization of LISA awakening phenotypes of mitochondrial and lysosomal mutants. a, Time-serial brightfield images showing behavioral recovery post-LISA in various strains indicated, including mitochondrial fusion-related *eat-3*, *fzo-1*, lysosomal dynamics-related *vps* and *hpo-27*, and mitophagy-related *dct-1* (LOF and GOF) and *pdr-1* mutants (n = 150 worms in each of 3 independent experiments). **b**, Population quantification of (a). **c**, Table listing a panel of strains to test the importance of mitochondrial fusion mutants and endo-lysosomal gene mutations in regulating the behavioral awakening from LISA. Two-way

ANOVA with Bonferroni post-test was used to compare mutant data with WT (**b**). *P* values are indicated.

Supplementary Data Legends

Supplementary Data 1.

RNAseq analysis of LISA. FPKM (Fragments Per Kilobase of transcript per Million mapped reads) and TPM (mapped reads) gene expression values of all *C. elegans* genes are shown for L4 control, control with mock recovery, LISA and LISA with recovery groups (three biological triplicates each).

Supplementary Data 2.

LC-MS analysis of LISA. LC-MS metabolite abundance data normalized to internal standards are shown and checked for quality control from control and LISA samples (four biological triplicates each).

Supplementary Data 3.

GC-MS analysis of LISA. GC-MS metabolite abundance data normalized to internal standards are shown and checked for quality control from control and LISA samples (four biological triplicates each).
