# Supplementary material for: Induction and regulation of reversible suspended animation in C. elegans
Source: Nat Commun. 2026 Mar 31;17:4627. doi: 10.1038/s41467-026-71247-9 (PMC13199508; doi:10.1038/s41467-026-71247-9)
Supplement: Supplementary file 2 — Description of Additional Supplementary Files [file 41467_2026_71247_MOESM2_ESM.pdf]

Title: Supplementary Data 1

Description: RNAseq analysis of LISA. FPKM (Fragments Per Kilobase of transcript per Million mapped reads) and TPM (mapped reads) gene expression values of all C. elegans genes are shown for L4 control, control with mock recovery, LISA and LISA with recovery groups (three biological triplicates each).

Title: Supplementary Data 2

Description: LC-MS analysis of LISA. LC-MS metabolite abundance data normalized to internal standards are shown and checked for quality control from control and LISA samples (four biological triplicates each).

Title: Supplementary Data 3

Description: GC-MS analysis of LISA. GC-MS metabolite abundance data normalized to internal standards are shown and checked for quality control from control and LISA samples (four biological triplicates each).
