# Supplementary material for: Induction and regulation of reversible suspended animation in C. elegans
Source: Nat Commun. 2026 Mar 31;17:4627. doi: 10.1038/s41467-026-71247-9 (PMC13199508; doi:10.1038/s41467-026-71247-9)
Supplement: Supplementary file 6 — Reporting Summary [file 41467_2026_71247_MOESM6_ESM.pdf]

nature portfolio

Corresponding author(s): Dengke Ma

Last updated by author(s): Mar 9, 2026

Reporting Summary

Nature Portfolio wishes to improve the reproducibility of the work that we publish. This form provides structure for consistency and transparency in reporting. For further information on Nature Portfolio policies, see our Editorial Policies and the Editorial Policy Checklist.

Statistics

For all statistical analyses, confirm that the following items are present in the figure legend, table legend, main text, or Methods section.

n/a	Confirmed	
[ ]	[x]	The exact sample size (*n*) for each experimental group/condition, given as a discrete number and unit of measurement
[ ]	[x]	A statement on whether measurements were taken from distinct samples or whether the same sample was measured repeatedly
[ ]	[x]	The statistical test(s) used AND whether they are one- or two-sided<br>*Only common tests should be described solely by name; describe more complex techniques in the Methods section.*
[x]	[ ]	A description of all covariates tested
[ ]	[x]	A description of any assumptions or corrections, such as tests of normality and adjustment for multiple comparisons
[ ]	[x]	A full description of the statistical parameters including central tendency (e.g. means) or other basic estimates (e.g. regression coefficient) AND variation (e.g. standard deviation) or associated estimates of uncertainty (e.g. confidence intervals)
[ ]	[x]	For null hypothesis testing, the test statistic (e.g. *F*, *t*, *r*) with confidence intervals, effect sizes, degrees of freedom and *P* value noted<br>*Give P values as exact values whenever suitable.*
[x]	[ ]	For Bayesian analysis, information on the choice of priors and Markov chain Monte Carlo settings
[x]	[ ]	For hierarchical and complex designs, identification of the appropriate level for tests and full reporting of outcomes
[x]	[ ]	Estimates of effect sizes (e.g. Cohen's *d*, Pearson's *r*), indicating how they were calculated

Our web collection on statistics for biologists contains articles on many of the points above.

Software and code

Policy information about availability of computer code

Data collection	Data were collected using instrument-specific acquisition software, including WormLab imaging software, Leica microscope software, Agilent MassHunter, and SCIEX Analyst.
Data analysis	GraphPad Prism 10 Software

For manuscripts utilizing custom algorithms or software that are central to the research but not yet described in published literature, software must be made available to editors and reviewers. We strongly encourage code deposition in a community repository (e.g. GitHub). See the Nature Portfolio guidelines for submitting code & software for further information.

Data

Policy information about availability of data

All manuscripts must include a data availability statement. This statement should provide the following information, where applicable:

- Accession codes, unique identifiers, or web links for publicly available datasets
- A description of any restrictions on data availability
- For clinical datasets or third party data, please ensure that the statement adheres to our policy

Source data are provided with this paper. RNA-seq data have been deposited in the Gene Expression Omnibus (GEO) under accession number GSE320035. All other data supporting the findings of this study, as well as reagents generated and/or analyzed during the current study, are provided and available from the corresponding author upon reasonable request.

Research involving human participants, their data, or biological material

Policy information about studies with human participants or human data. See also policy information about sex, gender (identity/presentation), and sexual orientation and race, ethnicity and racism.

Reporting on sex and gender	N/A
Reporting on race, ethnicity, or other socially relevant groupings	N/A
Population characteristics	N/A
Recruitment	N/A
Ethics oversight	N/A

Note that full information on the approval of the study protocol must also be provided in the manuscript.

Field-specific reporting

Please select the one below that is the best fit for your research. If you are not sure, read the appropriate sections before making your selection.

[x] Life sciences [ ] Behavioural & social sciences [ ] Ecological, evolutionary & environmental sciences

For a reference copy of the document with all sections, see nature.com/documents/nr-reporting-summary-flat.pdf

Life sciences study design

All studies must disclose on these points even when the disclosure is negative.

Sample size	Sample sizes were determined based on pilot studies and expert experience.
Data exclusions	No data was excluded in this study.
Replication	Most experiments were validated with repeat experiments at least 3 times.
Randomization	Animals were developmentally synchronized and allocated to experimental groups based on genotype or treatment condition. No formal randomization procedure was applied, as group assignment was predetermined by genotype or experimental manipulation. Experimental conditions were processed in parallel to minimize batch effects.
Blinding	Investigators were not blinded to group allocation during experiments. However, quantification was performed using predefined criteria and automated or standardized analysis workflows to minimize bias.

Reporting for specific materials, systems and methods

We require information from authors about some types of materials, experimental systems and methods used in many studies. Here, indicate whether each material, system or method listed is relevant to your study. If you are not sure if a list item applies to your research, read the appropriate section before selecting a response.

Materials & experimental systems

n/a	Involved in the study	
[ ]	[x]	Antibodies
[x]	[ ]	Eukaryotic cell lines
[x]	[ ]	Palaeontology and archaeology
[ ]	[x]	Animals and other organisms
[x]	[ ]	Clinical data
[ ]	[ ]	Dual use research of concern
[x]	[ ]	Plants

Methods

n/a	Involved in the study	
[x]	[ ]	ChIP-seq
[x]	[ ]	Flow cytometry
[x]	[ ]	MRI-based neuroimaging

Antibodies

Antibodies used	The following primary antibodies were used for western blotting:<br>Rabbit anti-GFP monoclonal antibody (MP Biomedicals, L100036, 1:10,000) and rabbit anti-β-tubulin polyclonal antibody (Proteintech, 10068-1-AP, 1:1,000). Antibodies were validated by the manufacturers, and validation data are available on the respective manufacturer websites.
Validation	All antibodies were commercially available and validated by manufacturers and/or citations. Manufacturer websites containing their validation data and citations, are listed below:<br>1) Rabbit anti GFP-Tag monoclonal antibody, MP Biomedicals, L100036, https://www.mpbio.com/us/rabbit-anti-gfp-tag-mab?queryID=bed9a68725b530309d23a8dca0d53343&objectID=56105&indexName=MpBio_Production_Store_us_products<br>2) Rabbit β-Tubulin Polyclonal antibody, Proteintech, 10068-1-AP, https://www.ptglab.com/products/TUBB3-Antibody-10068-1-AP.htm

Animals and other research organisms

Policy information about studies involving animals; ARRIVE guidelines recommended for reporting animal research, and Sex and Gender in Research

Laboratory animals	Caenorhabditis elegans (N2 Bristol background) were used in this study. Animals were maintained under standard laboratory conditions at 20 °C unless otherwise specified. Genotypes of all mutant and transgenic strains are listed in the Methods section. Unless otherwise indicated, experiments were performed using hermaphrodite animals. No vertebrate animals were used.
Wild animals	N/A
Reporting on sex	N/A
Field-collected samples	N/A
Ethics oversight	N/A

Note that full information on the approval of the study protocol must also be provided in the manuscript.

Plants

Seed stocks	N/A
Novel plant genotypes	N/A
Authentication	N/A
